# Can paraspinal muscle degeneration be a reason for refractures after percutaneous kyphoplasty? A magnetic resonance imaging observation

**DOI:** 10.1186/s13018-021-02623-y

**Published:** 2021-08-03

**Authors:** He Zhao, Yan He, Jun-Song Yang, Wei Bao, Jian Chen, Ji-Jun Liu, Qing-Da Li, Peng Liu, Bing Qian, Yuan-Ting Zhao, Ding-Jun Hao

**Affiliations:** 1 Department of Spine Surgery, Honghui Hospital, Xi’an Jiaotong University, No. 76 Nanguo Road, Xi’an, 710054 Shaanxi People’s Republic of China; 2 Department of emergency, Honghui Hospital, Xi’an Jiaotong University, No. 76 Nanguo Road, Xi’an, 710054 Shaanxi People’s Republic of China; 3 Department of Radiology, Honghui Hospital, Xi’an Jiaotong University, No. 76 Nanguo Road, Xi’an, 710054 Shaanxi People’s Republic of China; 4 Department of Orthopedics, People’s Hospital of Chongqing Banan District, Chongqing, People’s Republic of China; 5 Department of Orthopedics, Guolong Hospital, Yinchuan, Ningxia 750004 People’s Republic of China

**Keywords:** Osteoporosis, Vertebral compression fracture, Treatment, Vertebral fracture, Paraspinal muscle atrophy, Imaging modality

## Abstract

**Background:**

Vertebral augmentation (VA) techniques are used to treat acute osteoporotic vertebral compression fractures (OVCFs). However, the incidence of recurrent vertebral fractures after VA is controversial. Various factors have been discussed in the literature, but no convincing study on the quality of paraspinal muscles has been reported. The purposes of this study were to evaluate the changes in paraspinal muscles and discuss the relationship between paraspinal muscle degeneration and vertebral refractures after percutaneous kyphoplasty (PKP).

**Methods:**

This retrospective study was conducted in patients who underwent PKP for an initial OVCF between July 2017 and August 2018. Patients were followed up and categorized in the refractured or non-refractured group. A final magnetic resonance imaging (MRI) scan and a preoperative MRI scan were used to determine the measurements. The paraspinal muscles at the mid-height level of the initial fractured vertebral body were measured using regions of interest (ROIs), including the cross-sectional area (CSA) and signal intensity (SI). The changes in the observed data were compared between the groups using rank-sum tests.

**Results:**

Overall, 92 patients were enrolled in the study; 33 of them sustained vertebral refractures during the follow-up and the other 59 patients did not. There were no significant differences in terms of sex, age, preoperative bone mineral density, and body mass index between the groups (all, *P* > 0.05). The refractured group had a significantly higher decrease in the ROI-CSA and CSA/SI, and a higher increase in ROI-SI, compared with the preoperative data (all, *P* < 0.05).

**Conclusions:**

The quality of paraspinal muscles significantly decreased in patients with new OVCFs after PKP. This brings a new perspective to the study of postoperative recurrent fractures; patients and physicians need to pay more attention to the efficacy of bed rest and bracing.

## Background

Osteoporotic vertebral compression fractures (OVCFs) constitute a non-negligible senile problem, affecting 1.4 million patients in 2000 [1]. Percutaneous kyphoplasty (PKP) has been widely used for pain relief and vertebral stabilization, and it has been seen as safe and effective [2]. However, the long-term outcomes have become debatable concerning postoperative vertebral refractures [3–5]. Previous studies have shown that multiple vertebral fractures, bone cement distribution, and degree of kyphosis are associated with refracture after PKP [4]. Although PKP can provide kyphosis correction and height restoration, the holistic stability of the spine cannot be ignored. Finite element analysis confirmed that the quality of the back muscle was related to spinal stability [6]; a stronger back muscle may help prevent vertebral fractures in osteoporotic patients [7–9]. However, few previous studies have discussed the relationship between the back muscle mass and risk of new OVCFs postoperatively. Therefore, the purposes of this study were to evaluate the changes in paraspinal muscles and determine whether changes in the paraspinal muscle could be a reason for vertebral refractures after PKP.

## Methods

This retrospective study was conducted in patients who underwent PKP for an initial OVCF between July 2017 and August 2018. The informed consent was obtained from the patients. This study was performed in accordance with the Code of Ethics of the World Medical Association (Declaration of Helsinki) and was approved by the ethics committee of Honghui Hospital affiliated with Xi’an Jiaotong University.

All patients enrolled in this study met the following criteria: (1) patients initially diagnosed with single-level OVCF and agreed to undergo PKP, (2) available preoperative and final magnetic resonance imaging (MRI) data on the Picture Archiving and Communication System, and (3) complete medical information. The exclusion criteria were as follows: (1) patients with Kümmell disease, (2) a history of other spinal surgeries, (3) neurogenic disease or other activity disorder that may result in activity limitation, and (4) incomplete medical information.

**Fig. 1:**
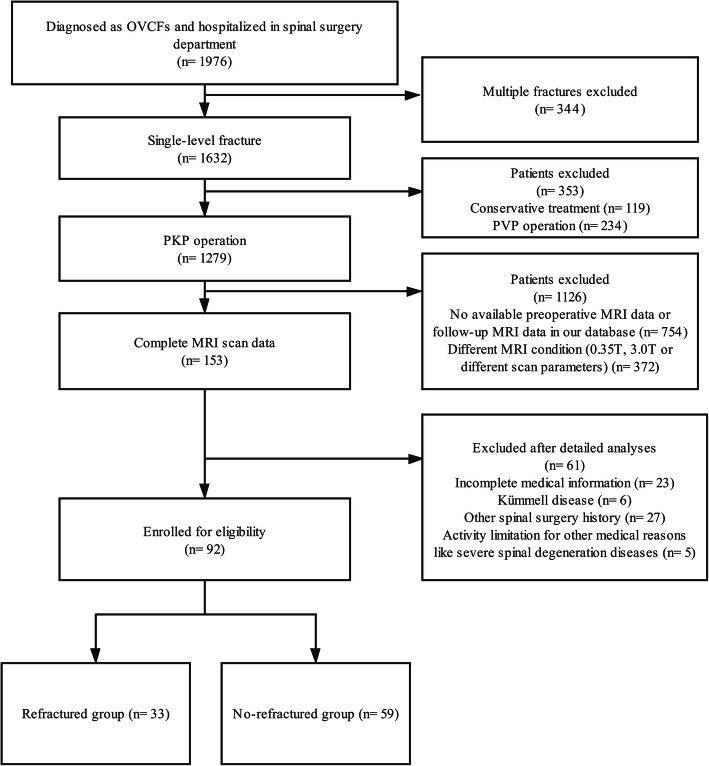
Flow diagram of participant screening, exclusion, and grouping

Patients with vertebral refractures, including augmented, adjacent, or nonadjacent vertebral bodies, returned to the hospital and their refractures were confirmed by MRI and other imaging examinations; these patients were categorized as the refractured group. Patients with no refracture or other acute injuries confirmed by an MRI scan in the outpatient follow-up were categorized as the non-refractured group. The final follow-up was performed at a minimum 3-month after PKP in both groups, and those who completed the follow-up qualified for enrollment (Fig. 1).

### Data collection and imaging measurements

**Fig. 2:**
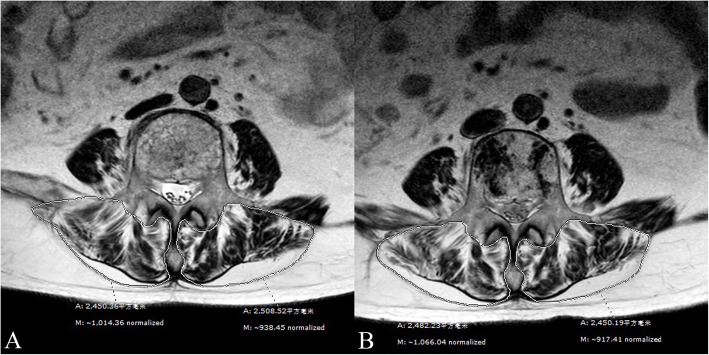
A 66-year-old woman with a OVCF at L4. **A** Preoperative axial T2-weighted image of L4 was measured. **B** Routine scan after 11 months reported no refracture occurrence. *A* areas, *M* mean grayscale

**Fig. 3:**
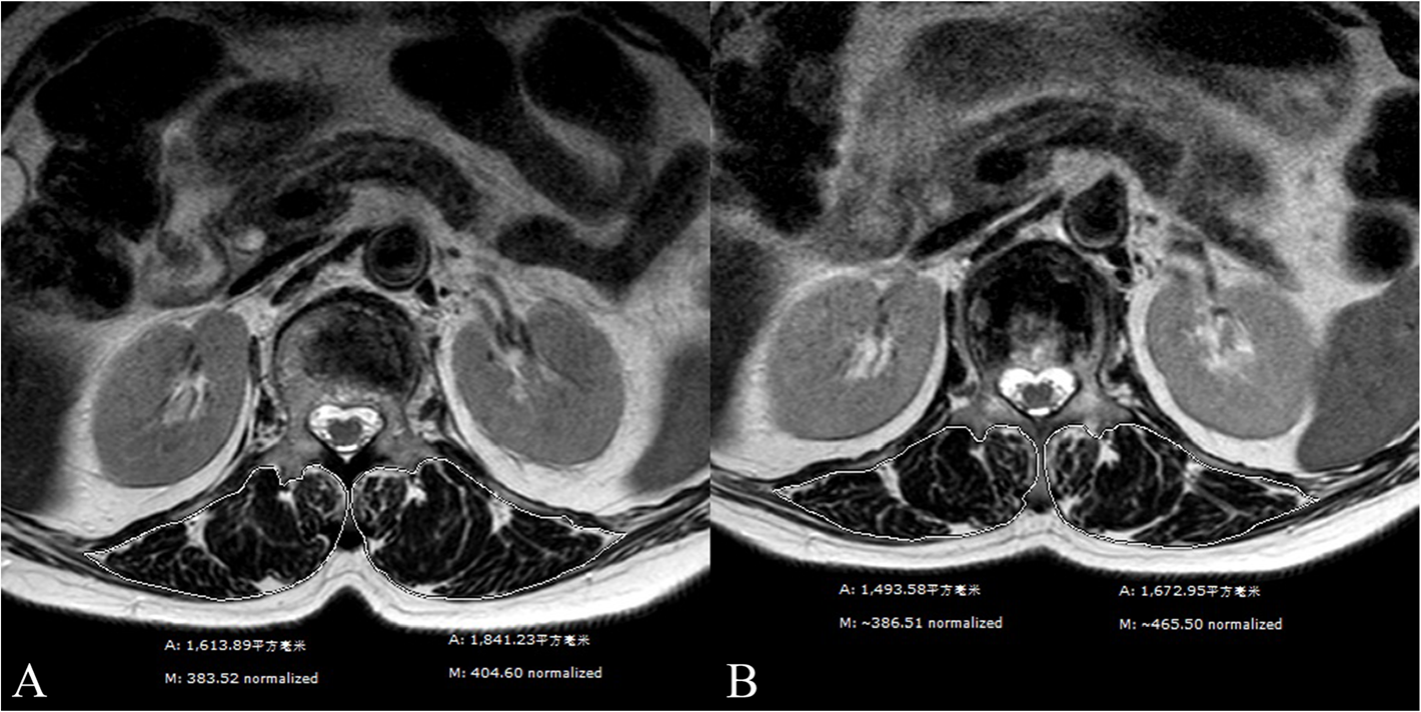
A 76-year-old woman with a OVCF at L1. **A** Preoperative axial T2-weighted image of L1. **B** Same image at L1 when T11 fractured after 7 months. The areas of paraspinal muscle decreased significantly. *A* areas, *M* mean grayscale

To evaluate changes of the paraspinal muscle between preoperatively and the final follow-up, MRI scan data (PHILIPS Achieva 1.5-Tesla unit, Amsterdam, the Netherlands; slice thickness, 4 mm; repetition time, 2206.52 ms; echo time, 120 ms) were analyzed using the Synapse 3D Workstation software program (FUJIFILM Medical System USA, Inc., Lexington, MA, USA). Patients examined with other MRI instruments or parameters were excluded. All paraspinal muscle measurements were conducted using a slice of the mid-height level of the initial fractured vertebral body on T2-weighted axial images. Both paraspinal muscles were manually contoured separately as the regions of interest (ROIs), as reported by Shahidi et al. [10] and, included the multifidus, erector spinae, and fat tissues inside the lumbosacral fascia posteriorly (Figs. 2 A, B and 3 A, B). Measurements were performed by two independent observers, including a spinal surgeon and an MRI specialist. The intraclass correlation coefficient (ICC) was used in the evaluation to ensure consistency.

The cross-sectional area (CSA) of the ROI was used to evaluate the size of the paraspinal muscle (mm^2^). Signal intensity (SI) was represented by the mean grayscale of the ROI quantified by the software, with higher scores indicating greater intensity. Wide edema, hemorrhage, and inflammation were excluded in consideration of measurement bias. Finally, the results of the observers were averaged. ROI-CSA and ROI-SI were recorded as the mean values of both muscle regions in each patient. The ratio of CSA/SI was analyzed, and it decreased as the muscles atrophied or more fat infiltrated them. Considering the wide differences among individuals, change in the aforementioned parameters between the two MRI scans was recorded as a percentage.

Demographic characteristics, including age, sex, surgical/refractory level, preoperative lumbar bone mineral density (BMD), body mass index (BMI), and duration of follow-up, were obtained from the Hospital Information System for further analyses.

### Statistical analyses

All statistical analyses were performed using Statistical Packages for Social Sciences version 21.0 (IBM Corp., Armonk, NY, USA). The evaluation of ICCs was conducted using a two-way random model with absolute agreement and an average measure (*κ* = 2). Measurement data were recorded by patient group. Sex was described as a dichotomous variable and analyzed using the chi-square test. The Shapiro-Wilk test was used to evaluate the distribution of quantitative variables; normally distributed variables are described as mean ± standard deviation, and then those were analyzed using the *t* test. Non-normally distributed variables are reported as median (quartile 1, quartile 3), and the rank-sum test (Mann-Whitney *U* test) was used to compare differences between the groups. Spearman correlation analyses were performed between the ROI-CSA and ROI-SI preoperatively and the percentage change separately. Statistical significance was set at *P* < 0.05.

## Results

Ninety-two patients (30 men and 62 women) who underwent PKP between July 2017 and August 2018 were enrolled in the study. The mean length of follow-up was 247.3 ± 90.5 days (range, 96–461 days). Thirty-three patients with newly developed OVCFs (range of segments, 1–3) were diagnosed by specialists. The other 59 patients who were followed up routinely were proven to have no refracture or similar injuries. The demographics are summarized in Table 1. There were no significant differences in sex distribution, age, preoperative BMD, and BMI between the refractured and non-refractured groups (*P* > 0.05).

**Table 1:** Comparison of demographic characteristics

	Groups		***χ***^**2**^/***Z***/***t***	***P***
	Refractured (***n*** = 33)	No-refractured (***n*** = 59)		
Gender (*n*)			0.012	0.912
Males	11 (33.3)	19 (32.2)		
Females	22 (66.7)	40 (67.8)		
Age (years)	74.27 ± 8.10	75.29 ± 8.51	0.558	0.578
BMI (kg/m^2^)	22.65 ± 2.11	22.55 ± 2.14	− 0.318	0.751
Preoperative BMD (T)	− 3.83 ± 0.63	− 3.65 ± 0.66	1.245	0.216
Follow-up duration (days)	215.12 ± 79.47	265.24 ± 91.90	/	

Categorical data was described as *n* (%); quantitative data were described as mean ± SD

Excellent interobserver agreements were demonstrated for all measurements, indicating the accuracy of the method. ICCs ranged from 0.898 to 0.954 for the following parameters: the left CSA (0.901; 95% confidence interval [CI] 0.782, 0.963), right CSA (0.898; 95% CI 0.771, 0.952), left SI (0.973; 95% CI 0.921, 0.986), and right SI (0.954; 95% CI 0.897, 0.977). The distribution of initial OVCF levels ranged from T5 to L5, and the measurements are shown in Table 2.

**Table 2:** The main measurements of paraspinal muscles

	Groups		***Z***/***t***	***P***
	Refractured (***n*** = 33)	No-refractured (***n*** = 59)		
**ROI-CSA** (**mm**^**2**^)				
Preoperative	1557.51 (1277.39, 1856.21)	1566.77 (1305.55, 1930.76)	− 0.094	0.925
Final follow-up	1432.54 (1105.15, 1718.53)	1512.38 (1278.10, 1970.32)	− 1.176	0.239
Percentage change (%)	− 10.1 (− 11.8, − 7.5)	− 1.3 (− 3.0, 0.2)	− 7.442	< 0.001
**ROI-SI**				
Preoperative	471.07 (404.33, 532.65)	466.52 (385.81, 579.83)	− 0.550	0.583
Final follow-up	529.31 (438.12, 597.54)	476.34 (402.25, 587.29)	− 1.221	0.222
Percentage change (%)	10.6 (5.3, 16.7)	1.4 (− 2.3, 4.1)	− 5.675	< 0.001
**The ratio of CSA/SI**				
Preoperative	3.38 ± 0.89	3.30 ± 0.88	0.412	0.681
Final follow-up	2.71 ± 0.72	3.23 ± 0.85	− 2.933	0.004
Percentage change (%)	− 19.2 (− 23.0, − 15.0)	− 2.5 (− 6.3, 0.5)	− 7.738	< 0.001

Normally distributed data were described as mean ± SD, while non-normally distributed data were reported by median (Q1, Q3)

### Cross-sectional areas

**Fig. 4:**
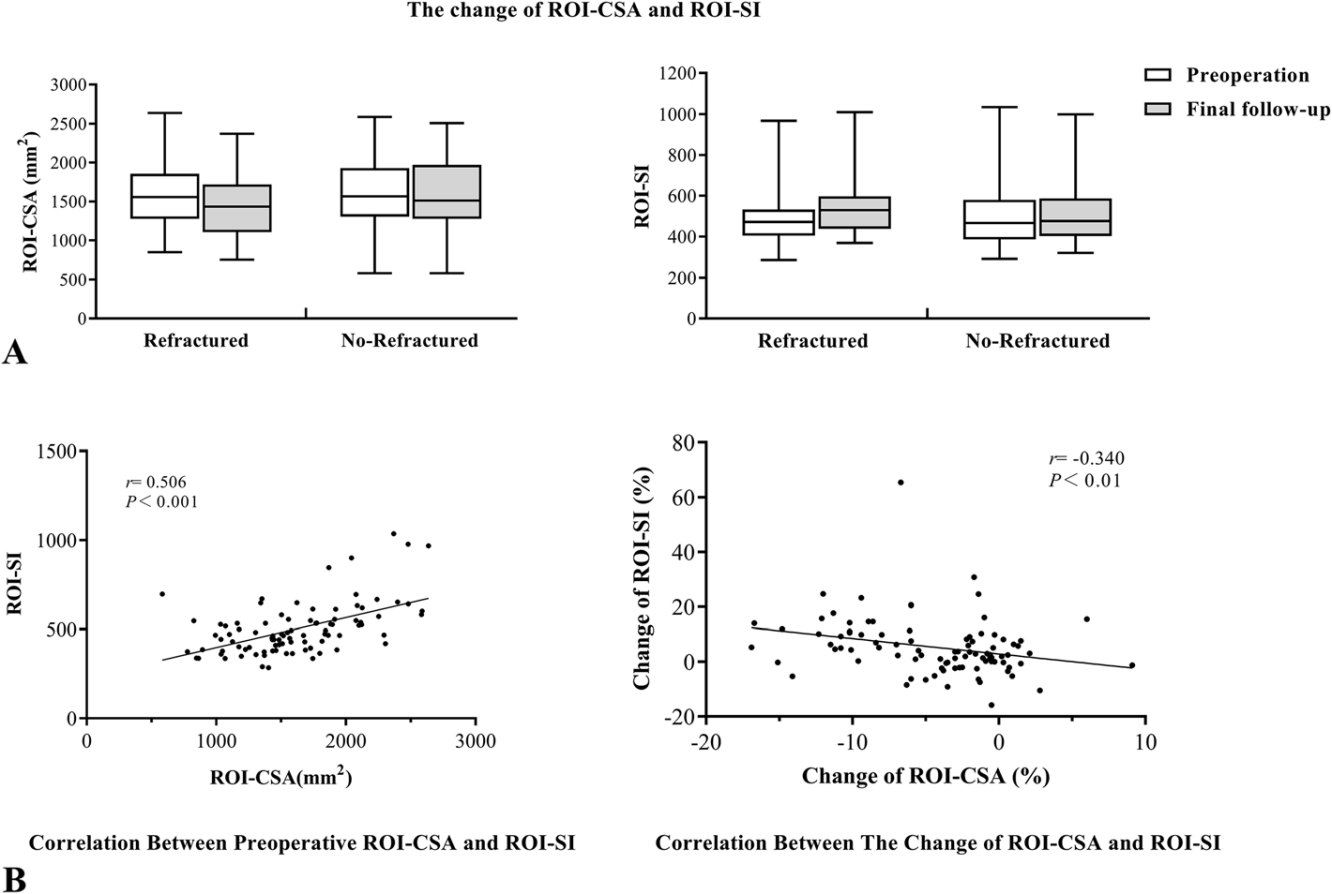
**A** The change of ROI-CSA and ROI-SI in two MRI scan measurements. **B** The consequences of correlation analyses conducted by Spearman test

Although the ROI-CSA had different degrees of reduction in all patients (Fig. 4A, B), there was no statistical difference between the preoperative and final groups (*P* > 0.05). The comparison of the percentage change in the ROI-CSA between the two MRI scans is shown in Table 2. The ROI-CSA in the refractured group decreased by 10.1%, which was significantly more than that the non-refractured group (1.3%) (*P* < 0.05).

The ROI-SI of both groups increased at the final follow-up (Fig. 4A, B). Patients with refractures had a slightly higher ROI-SI than those without refractures preoperatively; however, there was no statistically significant difference (*P* > 0.05). The change in the ROI-SI was a larger increase (10.6%) in the refractured group than in the non-refractured group (1.4%), and this was significantly different (*P* < 0.05).

Additionally, correlation analyses between the preoperative ROI-CSA and ROI-SI and the percentage change of the ROI-CSA and ROI-SI were performed, and both analyses showed significant correlations (Fig. 4A, B). Paraspinal muscles with a larger volume had a higher MRI SI preoperatively (*r* = 0.506, *P* < 0.001), and when the muscle became atrophied, the SI increased concomitantly (*r* = − 0.340, *P* < 0.01).

There was no statistically significant difference in the ratio of the CSA/SI preoperatively between the refractured and non-refractured groups, but the ratio of the refractured group decreased faster and showed a significant difference at the final follow-up (*P* < 0.05). When comparing the change in the CSA/SI, the ratio showed mean decreases of 19.2% and 2.5%, respectively, and the difference was significant (*P* < 0.05).

## Discussion

MRI is a noninvasive, radiation-free tool for determining muscle measurements, especially in terms of size and fat infiltration, and it has acceptable reliability [11]. In our study, to evaluate paraspinal muscle degeneration, we used the methods reported by Shahidi et al. [10]. Only 1.5-Tesla MRI was used in consideration of the sample size and image quality. Previous studies have affirmed the feasibility of performing measurements on axial MRI images [11–13]; however, any single segment of the paraspinal muscle measurement cannot be representative of the whole spine because of the significant difference in fat distribution [12]. Nevertheless, for patients with OVCF in our medical center, all MRI data included the axial image of the mid-height of the injured vertebral body. The measurements in this study were used to investigate the change in the fractured level of the paraspinal muscle using the same MRI scan parameters, thereby avoiding the errors caused by the uneven distribution of fat. With respect to measuring objects for the initial fractured segments ranging from T5 to L5, only the main composition of the paraspinal muscles was considered, except for the psoas, which was mentioned in studies on spinal degeneration and deformities. In view of these findings, we proposed that the outcomes could also represent the credible changes of paraspinal muscles in patients after PKP, and we hope to reflect the relationship between paraspinal muscle degeneration and the incidence of vertebral refractures.

PKP was performed only if the surgical principles were met. All patients were provided with a rigid thoracolumbosacral orthosis brace (TLSO, Hengshui Qianzhong Medical Equipment Co., Ltd., Hengshui, China) and were advised to undergo stabilization for 3 months (equal to the minimum follow-up criteria). All patients were instructed to attend regular follow-ups; 59 of them agreed to undergo another MRI scan and were proven to not have a refracture. Any case that did not meet the in/exclusion criteria was excluded.

After excluding unusual edema, hemorrhage, and inflammation, the ROI-SI can indirectly reflect the change in fat infiltration of the targeted muscles. Correlation analyses indicated that a stronger paraspinal muscle usually had a higher degree of fat infiltration, whereas the muscle atrophied along with fat infiltration (Fig. 4A, B). These results were similar to those of previous viewpoints [12] and supported the reliability of the methods used in measuring SI.

Spinal degeneration includes the vertebrae, discs, and joints and can be seen in muscles around the spine [6, 14]. Previous studies have reported changes in the morphology and composition of the muscles in adults. Fat infiltration in the paraspinal muscles has been found to increase progressively with age [10, 14, 15] and is more significant in the lower lumbar region than in the upper lumbar region [14, 16, 17]. In a long-term study, decreased muscle volume was also reported to be age-dependent in the normal population [15], and 15% of skeletal muscle mass was lost every 10 years in individuals older than 50 years of age [18]. In the present study, both patient groups showed a decrease in the ROI-CSA accompanied by an increase in the ROI-SI (Table 2). However, patients with refractures had significantly more paraspinal muscle shrinkage along with an increase in the SI compared to the non-refractured group. The CSA/SI ratios showed the same outcomes. In other words, patients after PKP were more inclined to sustain new OVCFs when the quality of the paraspinal muscle deteriorated.

Trunk muscles have been shown to be an important element of spinal stability. Biomechanical studies have reported that paraspinal muscles support the ligaments and vertebrae; therefore, weak paraspinal muscles would result in instability of the spine in patients with osteoporotic fractures. Inversely, stronger muscles can decrease stresses between the vertebrae, intravertebral discs, and facet joints and play an irreplaceable role at higher loads [6, 19].

The mass and density of muscle reduced yearly, thus limiting the daily mobility of elderly patients and leading to a higher risk of osteoporotic fractures [8, 20]. A case-control study of elderly men compared patients with lumbar fractures to healthy volunteers. At an average age of 75 years, the fractured group showed significantly lower CSAs and density in the paraspinal muscles as measured by quantitative computed tomography (CT) [20]. Research with a long follow-up reported that after performing a progressive back strengthening exercise for 2 years, the strength of the erector spinae muscle was enhanced in postmenopausal women and maintained for 10 years compared with the control group [8]. Moreover, people who performed a back muscle exercise had significantly fewer OVCFs in previous studies [8, 9], which was similar to the finding of our study.

Degeneration with aging was the main factor for muscle-bone unit dysfunction. Patients were inclined to have more bed rest or perform fewer daily activities after PKP, although their pain was relieved. The efficacy of prolonged bed rest is uncertain, and complications, such as muscle atrophy, atelectasis, thrombosis, and pressure ulcers, cause concerns [21]. Even short-term bed rest can induce acute bone resorption and decrease muscle strength [22]. Likewise, atrophied muscles and fat also affect one’s ability to perform activities, increase the risk of falling, and form a cycle of deterioration [23]. Patients with vertebral refractures usually were on bed rest for days before hospitalization; therefore, this might have been an unavoidable bias for the measurements in this study. However, the ROI-SI indeed increased significantly in these patients, and it required a long process.

Prolonged bracing treatment after OVCF is controversial. A brace is routinely used for stability after spinal fractures and surgeries. However, there is no strong evidence of the superiority of the brace after PKP; muscular atrophy was also suspected to be related to prolonged bracing. A randomized controlled trial reported that compared with the control condition, wearing a brace did not significantly help OVCF patients in terms of improving the Oswestry Disability Index scores or back pain [24]. Inversely, the drawbacks of bracing must be taken seriously. Reinforcing the trunk passively may lower the range of flexion and extension, subsequently leading to a reduction in paraspinal muscle strength [25, 26]. Ultrasound measurements showed that the lumbar muscles were atrophied after 8-week lumbar belt immobilization [27]. All patients in the current study were advised to use the TLSO for 3 months postoperatively. However, the specific processes, including duration and occasion, could not be recorded quantitatively. Based on the results, paraspinal muscle degeneration was seen in both groups, with the refractured group showing more significant deterioration overall than the non-refractured group. Disuse of paraspinal muscles was widespread in osteoporotic elderly patients, and we assumed that prolonged bracing and bed rest may be the components of vertebral refractures; however, no direct evidence of this has been presented yet.

According to the concept of the muscle-bone unit, appropriate loading forces of muscle could stimulate the formation of bone [20]. Conversely, lower BMD was also correlated with atrophic skeletal muscle [8, 20, 23]. To our knowledge, long-term low BMD was believed to be a prominent factor for OVCFs, and the preoperative BMD in the present study indicated a poor condition (Table 1). A BMI < 22 kg/m^2^ was mentioned in a previous study as a strong factor for predicting postoperative refractures [28]. A low BMI amplified the stress transferred to the adjacent vertebral bodies, which increased the possibility of new OVCFs [29]. Both groups showed similar BMIs and thus provided a comparable baseline; further multivariate analyses of BMD and BMI are required.

Medical injuries of the paraspinal muscles have to be considered. The PKP technique uses trocars, and it is believed to be minimally invasive compared with the open approach. However, damage to the paraspinal muscle is inescapable. In addition, procedures were more difficult to perform in cases of severe degeneration, and repeated adjustment of the needles was related to more damage to the muscles and branch nerves. The unilateral approach causes less muscular injury than bilateral injections, but there is no study on muscular evaluation. It seemed unavoidable that the ROI-SI increases with fat and edema when using T2-weighted images. However, OVCFs in elderly patients were mostly caused by moderate trauma, and patients with obvious muscle edema and inflammation were excluded, which make the results more tenable.

Based on the results of this study, we have discussed the relationship between vertebral fractures and paraspinal muscle degeneration in elderly individuals. In the context of low BMD, people were prone to new OVCFs, but weak paraspinal muscles may also be a risk factor. In clinical practice, prolonged bed rest and bracing treatment are worthy of concern. We hope that this MRI observation could provide a new perspective on refractures after PKP. In addition, a comparison of paraspinal muscles between patients with OVCFs and healthy elderly people is required.

Several limitations of this study need to be noted. First, it was a retrospective study that lacked substantial samples. Only patients with complete 1.5-Tesla MRI data were enrolled to ensure consistency. Second, the final follow-up MRI in the non-refractured group was not entirely random, and potential bias should be mentioned. Furthermore, using a grayscale to estimate SI is an indirect method, although its reliability is acceptable. Further studies using quantitative CT and ultrasound approaches will be more precise.

## Conclusions

Our data showed that the quality of the paraspinal muscle significantly decreased in patients with new OVCFs after PKP. This brings a new perspective to the study of postoperative recurrent fractures; patients and physicians need to pay more attention to the efficacy of bed rest and bracing.

## Data Availability

The datasets used and/or analyzed during the current study are available from the corresponding author on reasonable request.

## Abbreviations

VA: Vertebral augmentation
OVCFs: Compression fractures
PKP: Percutaneous kyphoplasty
MRI: Magnetic resonance imaging
ROIs: Regions of interest
CSA: Cross-sectional area
SI: Signal intensity
BMD: Bone mineral density
BMI: Body mass index
